# Research on the localized lightfield features of metallic nano-cone-tip optical antenna via investigating near-field lightwave and correlated net-charge distribution

**DOI:** 10.1038/s41598-023-49097-y

**Published:** 2023-12-12

**Authors:** Taige Liu, Jiashuo Shi, Shenghua Duan, Wuyang Ji, Zhe Wang, Xinyu Zhang

**Affiliations:** 1 National Key Laboratory of Multispectral Information Intelligent Processing Technology, Huazhong University of Science and Technology, Wuhan, 430074 China; 2 School of Automation and Artificial Intelligence, Huazhong University of Science and Technology, Wuhan, 430074 China

**Keywords:** Nanoparticles, Quantum information, Nanophotonics and plasmonics

## Abstract

In this paper, the near-field lightwave characteristics of an arrayed silicon nano-cone-tip optical antenna (NOA) covered by a common metal film, which can be viewed as a featured quasi quantum dot (QD), are carefully investigated. A dipole net-charge distribution closely correlated with the surface lightwaves excited over the antennas by incident lasers with a central wavelength of 633 nm, is clearly observed. An obvious Coulomb-like blockade from the apex apparently influencing the net-charge converging over the surface of NOA, is verified, which can also be predicted by the simulations according to surface standing waves across the apex node. The antinodes of the surface net-charge instantaneous distribution are already pushed away from the normal location owing to the apex Coulomb-like blockade, so as to present a distorted waveform different from traditional standing wave modes. The tip proximity effect leading to a relatively weak net-charge converging over surrounding planar facet and adjacent NOAs, is also discovered.

## Introduction

In recent years, multi-types special functioned micro-nano-architectures demonstrate several significant features in efficiently adjusting or even modulating physical properties via strongly coupled with incident electromagnetic radiations in a relatively wide wavelength range, and thus highlight various potential applications in medical imaging, biosensor, surface enhanced Raman spectroscopy, and fluorescence imaging^1–7^. An arrayed nano-tip, as a typical periodic an optical antenna providing a large number of tip surface states, has been successfully constructed based on many common metal materials, so as to predict a possibility through driving or compressing a large number of surface “free electrons” onto the sharp apex of a single tip, and thus results in an extremely strong surface “free electron” nano-concentration over the apex. The accumulation of surface “free electrons” will directly results in a highly localized enhancement of both the resonant electric-field and magnetic-field components converged or even focused at apex.

Thanks to a relatively strong response and then highly localized resonant enhancement, the surface waves with the featured frequencies can be effectively excited and then transported towards the apex along tip surface under the constraint of the tip boundary after continuous refraction and reflection. Then, the local light wave amplitude will get significantly enhanced, which allows for the efficient conversion of propagating light irradiation into strongly enhanced optical field confined in nanoscale, and vice versa. Currently, a remarkable enhancement of the near-field electric- or magnetic-amplitude or intensity allows an amazing extent of exceeding 6 orders of magnitude through converging or even focusing incident radiations into a nanoscale space^8–13^. According to the previous research, an efficient conversion from propagating wavefields to highly localized lightfields is mainly determined or constrained by surface net-charge such as “free electron” distribution behavior. The incident lightwaves can be resonantly amplified based on the typical nano-converging or even nano-focusing of nano-tips^14–16^. A number of research based on highly compressed surface “free electrons” onto the apex of a single metal tip excited by incident laser pulses, so as to realize a near-field probing, has been proposed. The detection sensitivity based on colloid quantum dots (QDs) can be remarkably improved according to nanosecond or even attosecond measurements^17–22^. However, the detailed research about the dynamic transportation and redistribution of surface “free electrons” within the nanoscale region, as well as the propagating property of the stimulated surface wave over the tip-typed antennas still remains limited.

The significant quantum property, especially the Coulomb blockade, has been observed in InAs nanowire segment^23–25^ and nanoball-shaped droplet semi-conductor QD^26,27^. Analyzing the dynamic re-arrangement properties of the surface “free electrons” towards the apex of a single nano-tip, which can be viewed as a special quasi QD, will enable a remarkable optimization of the nano-converging or even nano-focusing performance. Generally, a high density of energy level distribution determined by the wave functions of “free electrons” or holes confined into a nanoscale space or geometric point, can stimulate surface exciton and further drive the free electrons tunneling resonantly into quasi QDs^17,28–31^. As demonstrated, the electronic distribution probability over the surface of quasi QDs can be considerably boost due to tunneling or exciton effect, which is closely related with the electron occupation characteristics of the apex surface state. At the same time, a flexible adjustability of band gap via changing the architectural properties or even an effective modulation of radiation intensity, are the significant advantages of quasi QDs, which makes them highly promising materials for near-field nano-converging or even nano-focusing^32–34^.

In this paper, the theoretical models about the key electronic and near-field lightwave characteristics of an arrayed nano-cone-tip optical antenna (NOA), are constructed. The numerical simulations indicate that the net-charges such as surface “free electrons” rarely reach the apex but usually distribute in lower area of the NOA under a relatively strong beam illumination or surface wavefield excitation, due to a great Coulomb-like blocking of the net-charges over the apex, which remarkably restrain other net-charges continuously into the limited apex region, and thus forms surface near-field waves in a standing wave mode with a featured waveform across the apex node. In order to validate the simulation, a near-field measurement corresponding to an arrayed NOA covered by common metal materials is performed according to the scattering-type scanning nearfield optical microscopy (SNOM), which directly present a character that the near-field lightwave excited by incident laser beams with a central wavelength of 633 nm is closely related with the surface net-charge morphology. The situation of remarkably enhancing near-field lightwaves also means a strong surface net-charge accumulation not only around the apex facet, but also over the surrounding planar facet and adjacent NOAs. The lightwave transmission based on the redistribution of the surface “free electrons” can still be explained according to the typical characteristics of quasi QDs.

## Results and discussion

### Numerical simulation

A series of numerical simulations about a dipole surface net-charge distribution over an arrayed metallic NOA with typical features including the nano-geometry and the structural material, are carried out by finite difference time domain method. A periodic boundary condition of 500 nm in both x and y directions, and perfectly matched layer (PML) in z direction, are utilized for the FDTD simulation. An arrayed NOA has been designed and fabricated over a monocrystal silicon wafer, and a gold film with a thickness of 30 nm continuously deposited over its surface, so as to introduce a dense surface state over the NOAs^29^. Then, the net-charge instantaneous distribution morphologies around the apex of a single metallic NOA mainly stimulated by incident laser beams, are further obtained. The net-charge density over the NOA surface is carefully analyzed according to common Maxwell’s equations and then particularly based on a key relation of $\rho e = \nabla \cdot {\mathbf{D}}$. Consequently, a surface electric-field component that is tightly constrained by the surface net-charges stimulated and points outside the NOA surface, can be expressed as a z-component *E*_*z*_. Owing to the simultaneous acquisition of both the amplitude $Sj(x,y,z,\theta ,d)$ and the phase $\varphi j(x,y,z,\theta ,d)$ of ***E***_***z***_, the net-charge density $fj(x,y,z)$ at any position (*x, y, z*) of the NOA can be represented by an equation^8^ of

1 $$ fj(x,y,z) = {\text{Re}} [Sj(x,y,z,\theta ,d)e^{{{\text{i}}\varphi j(x,y,z,\theta ,d)}} ] $$

where *d* and *θ* are the bottom diameter and the top angle of a single NOA, respectively, and *j* (= 1, 2, 3, …) represents the metal film material already deposited over the NOA surface, for example, *j* = 7 means Au. So, the metallic NOAs can be characterized according to the structural parameters of the bottom diameter *d* and the top angle *θ*, and then investigate the surface net-charge resonating distribution involving the net positive charge and the aggregated “free electrons”.

**Figure 1:**
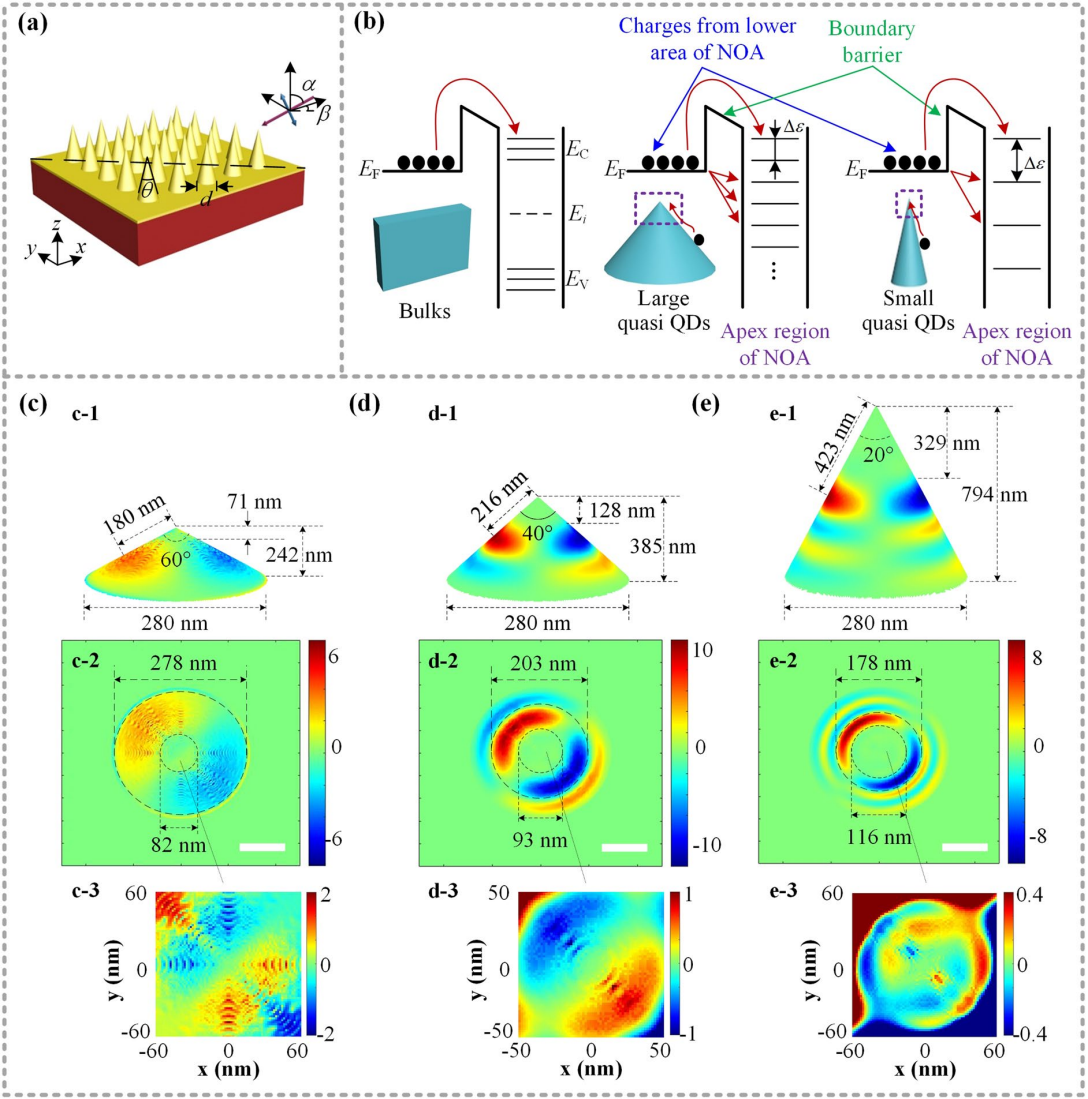
Simulating the surface charge resonating distribution of metallic NOAs with different top angle *θ.* (**a**) An arrayed metal NOA illuminated by an incident beam with a central wavelength of ~ 633 nm and an initial polarization labeled by a double-sided arrow along an orientation of (*α*, *β*). (**b**) Energy level diagrams of typical semiconductors from bulk materials to tip-typed quasi QDs with different geometry. Typical net-charge instantaneous distribution over a single NOA with different parameters: (**c**) *d* = 280 nm, *θ* = 60°, (**d**) *d* = 280 nm, *θ* = 40°, (**e**) *d* = 280 nm, *θ* = 20°. The 3D simulations are exhibited by (**c-1**) and (**d-1**) and (**e-1**), and the main DNCS shaped by a couple of net positive charges and aggregated “free electrons” corresponding to a pixel area of 500 nm × 500 nm (scalar bar 100 nm) are shown in (**c-2**) and (**d-2**) and (**e-2**), respectively. Three enlarged viewings of the patterned dipole net-charge instantaneous distribution at each apex region are also presented in (**c-3**) to (**e-3**). Noting that the positive and negative signs of the right color-bars represent the positive net-charges and the aggregated “free electrons”. The adjustable upper and lower diameters of each main DNCS are roughly marked by long and short dashed circles. See details in Fig. S1 in the Supporting Information.

**Figure 2:**
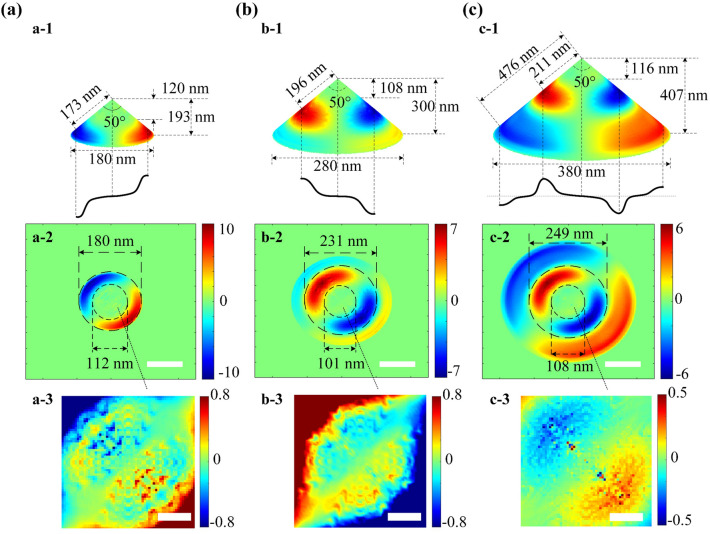
Typical net-charge instantaneous distribution over the surface of a single NOA with the same top angle of 50° but different bottom diameter *d*: (**a**) 180 nm, (**b**) 280 nm, and (**c**) 380 nm. The 3D simulations are shown in (**a-1**) to (**c-1**), where the key structural parameters about the main DNCS based on the standing wave mode having the same apex node and the featured schematic waveform are also given. The top views of the net-charge distribution corresponding to a pixel area of 500 nm × 500 nm are exhibited in (**a-2**) to (**c-2**) (scalar bar 100 nm), where the upper and lower boundary circles of the main DNCS are indicated by both the long and short dashed circles, respectively. The enlarged viewings of the patterned net-charge over the apex region sized in 100 nm × 100 nm are shown in (**a-3**) to (**c-3**) (scalar bar 30 nm).

The surface net-charge distribution according to the configurated beam illumination over metallic NOA arrays, are simulated as shown in Fig. 1. A beam of visible lightwave with a central wavelength of ~ 633 nm and an initial polarization labeled by a double-sided arrow, is incident obliquely along the diagonal with an incident angle of 45° upon a horizontal facet, that is $\alpha = \beta = 45^{ \circ }$, as shown in Fig. 1a. The energy level diagrams of the typical semiconductors from bulk materials to tip quasi QDs with different geometry is shown in Fig. 1b. Different with bulk material, the apex surface states, originated from the discrete valence band or conduction band, as well as the acceptor energy levels in the forbidden band, will become substantial without an obvious forbidden band as typical QDs The ways for charges from lower area of NOA (labeled by blue arrows in Fig. 1b) to get into apex region with higher energy state include two mechanisms: (1) absorb sufficient external energy to form exciton, so as to jump over the boundary potential barrier (labeled by green arrows in Fig. 1b); (2) tunnel into apex region to avoid the barrier, which is probable to happen in nanoscale. Note that the boundary potential barrier is positioned within the NOA fabricated with single material to separate the regions with higher energy state and lower one. The NOA provides ample unoccupied level, so as to form a highly charged facet or even guide net-charge tunneling into tip quasi QDs instead of crossing the boundary barrier^35,36^. Moreover, the trend of gradually shrinking NOA, which means that the apex of NOA will become sharper, will bring a wider band gap and lower density of unoccupied tip surface states to restrain more net-charge into the apex. As a result, the possibility of the net-charge filling behavior will be decreased apparently.

As shown in Fig. 1c–e, keep the NOA bottom diameter of 280 nm but gradually decrease the top angle *θ* from 60° to 20°. In other words, the height of the NOA will be increased from ~ 242 to ~ 794 nm, but having a constant bottom size. So, the main dipole net-charge converging spot (DNCS) presents a trend of being away gradually from the apex, where the height between the apex and the upper circle of the main DNCS will be remarkably increased from ~ 71 to ~ 329 nm, during continuously sharping the NOA, so as to increase its height, as shown in Fig. 1c-1 to e-1. See more details in Fig. S1 in the Supplementary Information. Note that the tip-type quasi QD refers to a particular nano-district or nano-area with relatively low distribution density, specifically below 0.1 unit instead of the apex defined the bottom diameter as the upper circle of the DNCS, as illustrated in Figs. S2 and S3 in the Supplementary Information. Consequently, an apparent width narrowing of the main DNCS enclosed between the upper long dashed and lower short dashed circles, can be viewed in a sequence of ~ 196 to ~ 62 nm. Based on the surface net-charge instantaneous distribution simulations mentioned, an obvious Coulomb-like blockade phenomenon, the net-charge lacking over the nanoscale apex region, and splitting on the opposite side of NOA with a dipole distribution, can be clearly observed. The distance between the central arc of a main DNCS and the apex presents a sequence of ~ 180 nm to ~ 216 nm to ~ 423 nm. Considering the case that the surface net-charge resonant distribution is closely related with the surface near-field lightwaves, which should be a standing wave with a central node at the apex. Moreover, the center-to-center distance between the positive and the negative net-charged regions of the main DNCS along NOA surface is defined as a half-wavelength of the surface wave stimulated by the incident beams. It should be noted that the half-wavelength of the stimulated surface waves corresponding to the NOAs with different structural size is 360 nm and 432 nm and 846 nm, respectively, which are greater than incident beams.

**Figure 3:**
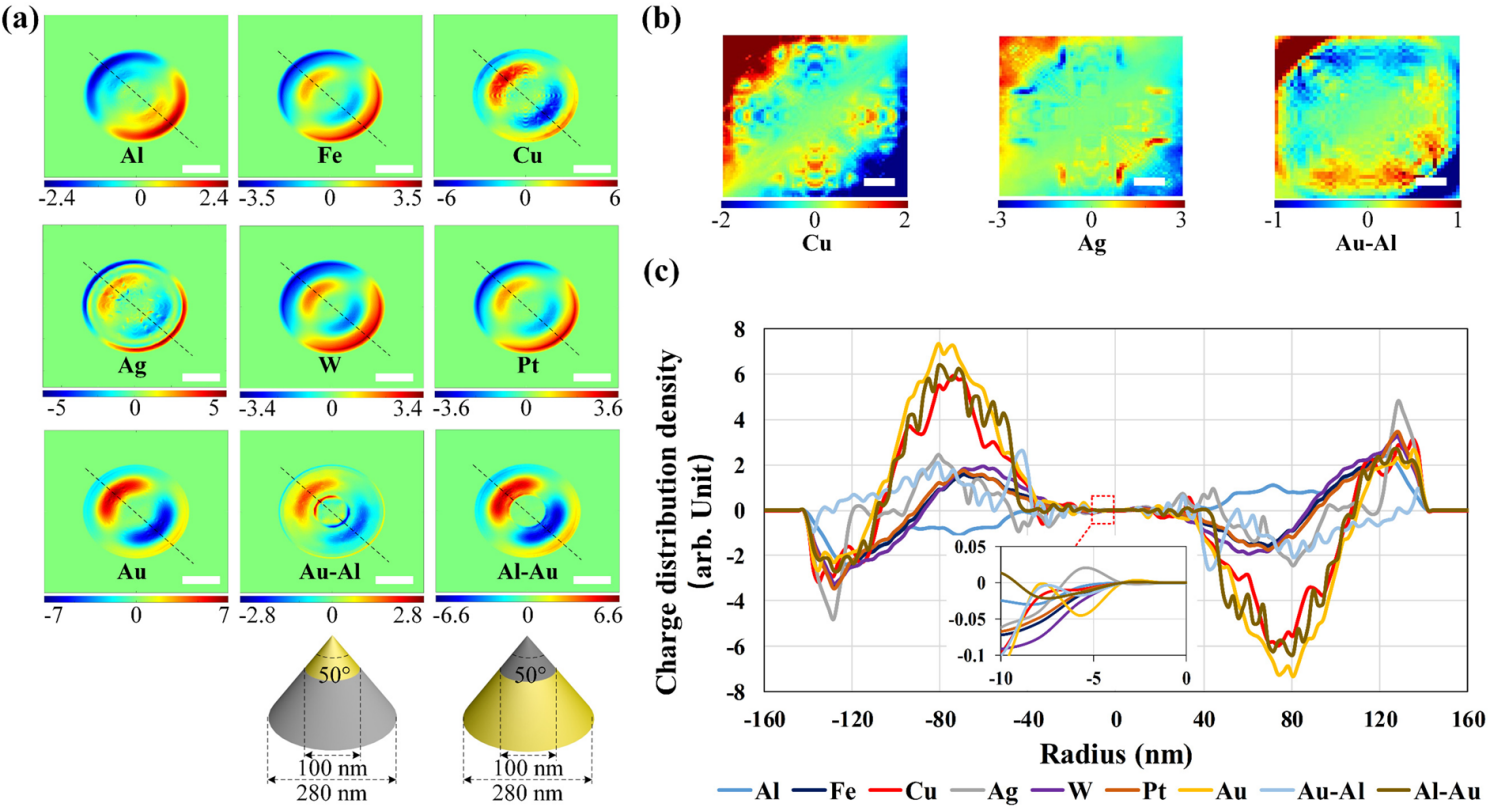
Comparison of the net-charge instantaneous distribution of the NOAs with the same bottom diameter of 280 nm and the top angle of 50° but precoated by different metallic materials including Al, Fe, Cu, Ag, W, Pt, Au, Al-Au, and Au-Al. (**a**) The dipole net-charge instantaneous distribution from the top viewing within a pixel area of 500 nm × 500 nm (scalar bar 100 nm). The structural diagram based on the Au-Al and Al-Au heterofilms are also given. (**b**) The enlarged viewing of the patterned net-charge over the apex sized in an area of 120 nm × 120 nm covered by Cu, Ag, and Au-Al (scalar bar 20 nm). (**c**) The net-charge density curves along a diagonal marked by a black dashed line in (**a**), where the net-charge density in a range from − 10 nm to 0 is also inserted.

As shown in Fig. 1c-2, the positive net-charge and the aggregated “free electrons” are respectively colored by pale yellow and light blue. Moreover, the instantaneous main DNCS are confined between a long-dashed circle with ~ 82 nm diameter and a short-dashed circle with ~ 278 nm diameter, and also distribute separately over opposite side of a single NOA with a top angle of 60°. The distinct distribution of net-charges over the opposite side of the NOA can attribute to a relatively strong interference of the incident beams and the surface waves stimulated. And the interference results in an intensity variance from − 6 to 6 unit according to the color-bar. An enlargement viewing of the patterned net-charges over the apex region with a relatively low intensity in a range from − 2 to 2 unit is further exhibited in Fig. 1c-3. Similar to the above simulation results the net-charges are rarely concentrated over the top region around the apex node, and split into a symmetrical distribution fringes along the diagonal of NOA. These fringes consist of alternating arrangement of the positive and negative net-charges adjacent to the main DNCS, and distribute over the opposite side. As a very small apex capacitance strongly restraining the net-charge climbing into the apex, the Coulomb-like blockade of quasi QDs^37–39^ will get enhanced through decreasing the top angle *θ* from initial 60° to medial 40° to final 20°, and thus the distance between the central arc of a main DNCS and the apex being rapidly increased, where the maximum net-charge density is from 2 to 1 to 0.4 unit, as illustrated in Fig. 1c-3 to e-3.

Several typical net-charge instantaneous distribution over a single NOA with the same top angle of 50° but different bottom diameter *d*: (a) 180 nm and (b) 280 nm and (c) 380 nm, which corresponds to the different NOA height including ~ 193 nm and ~ 300 nm and ~ 407 nm, are simulated, as illustrated in Fig. 2. The main DNCSs are also confined between a long-dashed circle with ~ 180 nm diameter and a short-dashed circle with ~ 112 nm diameter, and thus distribute separately over its opposite side when *d* = 180 nm, as shown in Fig. 2a-2. And an approximate distance of ~ 173 nm between the central arc of a DNCS and the apex can be obtained, as shown in Fig. 2a-1. A typical wavelength of approximately 692 nm can be determined to the shaped surface standing wave, which is closely associated with the distribution of positive and negative net-charges around the apex. This wavelength is slightly longer than the incident wavelength of 633 nm, due to a relatively weak Coulomb-like blockage of similar dipole net charges in the apex region, as depicted in Fig. 2a-3. This finding aligns with the observed trends in both distance and net-charge variation illustrated in Fig. 1. It should be noted that the small bottom diameter of 180 nm means a NOA boundary at the bottom, which also slightly hinders the net-charge re-arrangement away the apex and then nearing the bottom boundary. Consequently, a synergistic compression effect is observed, due to the combination of the apex Coulomb-like blockade and the bottom boundary constraint.

As increasing the height but remain *θ* of NOA, the upper arc of the main DNCS slightly decreases to ~ 101 nm, whereas the lower one increases to ~ 231 nm, as shown in Fig. 2b. Then, a widened size of the main DNCS from ~ 68 to ~ 130 nm can be observed. A slight decreasing of the net-charge instantaneous density from 10 to 7 unit according to the color-bar, can be predicted, as shown in Fig. 2b-2. And then the surface standing wave demonstrates a half-wavelength of ~ 393 nm, which is larger than that of *d* = 180 nm, because of a relatively weak bottom boundary constraint but a similar Coulomb-like blockade action of the apex. Figure 2c-1 shows a surface standing wave mode with two symmetrical nodes on both sides of the NOA and an apex node, where both positive and negative second net-charge converging spots are at the bottom boundary after increasing *d* to 380 nm. The distance between the edge DNCSs along the NOA and across the apex node corresponding to 1.5 wavelength is ~ 952 nm, which means that the surface standing wave with a wavelength of 634.7 nm. This wavelength is almost identical to that of incident beams.

The numerical simulation about the distance relation provides a direct evidence that the Coulomb-like blockade action will rapidly decay away from the apex, and finally be neglected when the distance being increased to ~ 476 nm. Additionally, the height difference between the apex and the upper dashed circle of the main DNCS (~ 120 nm, ~ 108 nm, and ~ 116 nm) suggests that the net-charge around the apex with a given top angle will be influenced by a similar Coulomb-like blockage effect, and then a distorted standing waveform. The maximum net-charge density is positioned almost the same in the cases of *d* = 280 nm and 380 nm according to the same Coulomb-like blockage action. As demonstrated in Fig. 2a-3,b-3,c-3, the dipole net-charge over the apex with various bottom diameter has a comparable distribution pattern and intensity.

Additionally, how the metal materials coated over the silicon NOA influences the net-charge resonant distribution, is also studied carefully. As shown in Fig. 3, several NOAs all shaped in *d* = 280 nm and *θ* = 50° are simulated. Each NOA is continuously coated by different metal materials labeled by a subscript *j* in Eq. (1) from 1 to 7, including Al, Fe, Cu, Ag, W, Pt and Au. The apex region above a 100-nm demarcation diameter and the lower frustum side surface are coated with different metals to form a heterofilm over the NOA surface. The chosen sequence involves applying top Au to frustum Al, as well as top Al to frustum Au. This selection is based on the significant disparity in atomic number and work function between these metals. Figure 3a demonstrates a relatively obvious net-charge instantaneous distribution over the apex in case of covered by Cu, Ag, Au and Al-Au, down to 5 unit. Meanwhile, the net-charge instantaneous distribution over the apex of the NOAs covered by Al and Fe and W and Pt and Au-Al, respectively, are relatively weak compared to above according to the color-bar with a maximum value of 2.4 unit. As shown, the net-charge density of the NOAs covered by different heterofilm of Al-Au and Au-Al exhibit a relatively large variance of more than twice, due to the work function difference between Al and Au, which are commonly 4.28 eV and 5.1 eV, respectively.

Several enlarged viewings of the DNCS distributed over the apex, which demonstrate almost the same net-charge distribution pattern with a similar density variance range, are given in Fig. 3b. As shown, the dipole net-charges over the apex region of a single NOA covered by Cu and Ag and Au-Al film, respectively, are also symmetrical about the apex node, and all present a relatively high density in an arrange from − 3 to 3 unit. In addition, the net-charge density along a diagonal marked by a black dashed line, as shown in Fig. 3a, is further plotted in Fig. 3c, where the x-axis represents the cross-sectional radius of the NOA and the zero point the apex, so as to fully display the surface net-charge instantaneous arrangement character. An enlargement view indicating initial divergence of the curves away the apex node in a range from − 10 to 0 nm is also inserted. The net-charge density obviously presents a point symmetry about the apex node. The peak value corresponding to Au film reaches ~ 7 unit at − 80 nm radius of the central arc of the DNCS and also approach the apex node more closely. In contrast, Al film exhibits the worst net-charge convergence with the lowest net-charge distribution density up to ~ 2.4 unit and the furthest distance between the main DNCS and the apex, which may due to the lowest atomic number and the relatively low work function.

As shown, the net-charge instantaneous arrangement over the NOA precoated by common metal film is quantitatively simulated. An obvious variance between a silicon Al-NOA with a maximum value of 2.4 unit and a silicon Au-NOA with a maximum value of 7 unit, can be observed. After shaping an Au-Al heterofilm, an obvious density decreasing of the main DNCS from ~ 7 to ~ 2.8 unit can be seen, because the main DNCS being over the surface of Al film. An almost unchanged density of the main DNCS around ~ 7 unit mainly distributed over Au film, can be viewed after shaping an Al-Au heterofilm. So, a rapid density increasing of the main DNCS can be realized through reasonably configurating metal heterofilm over the same NOA.

**Figure 4:**
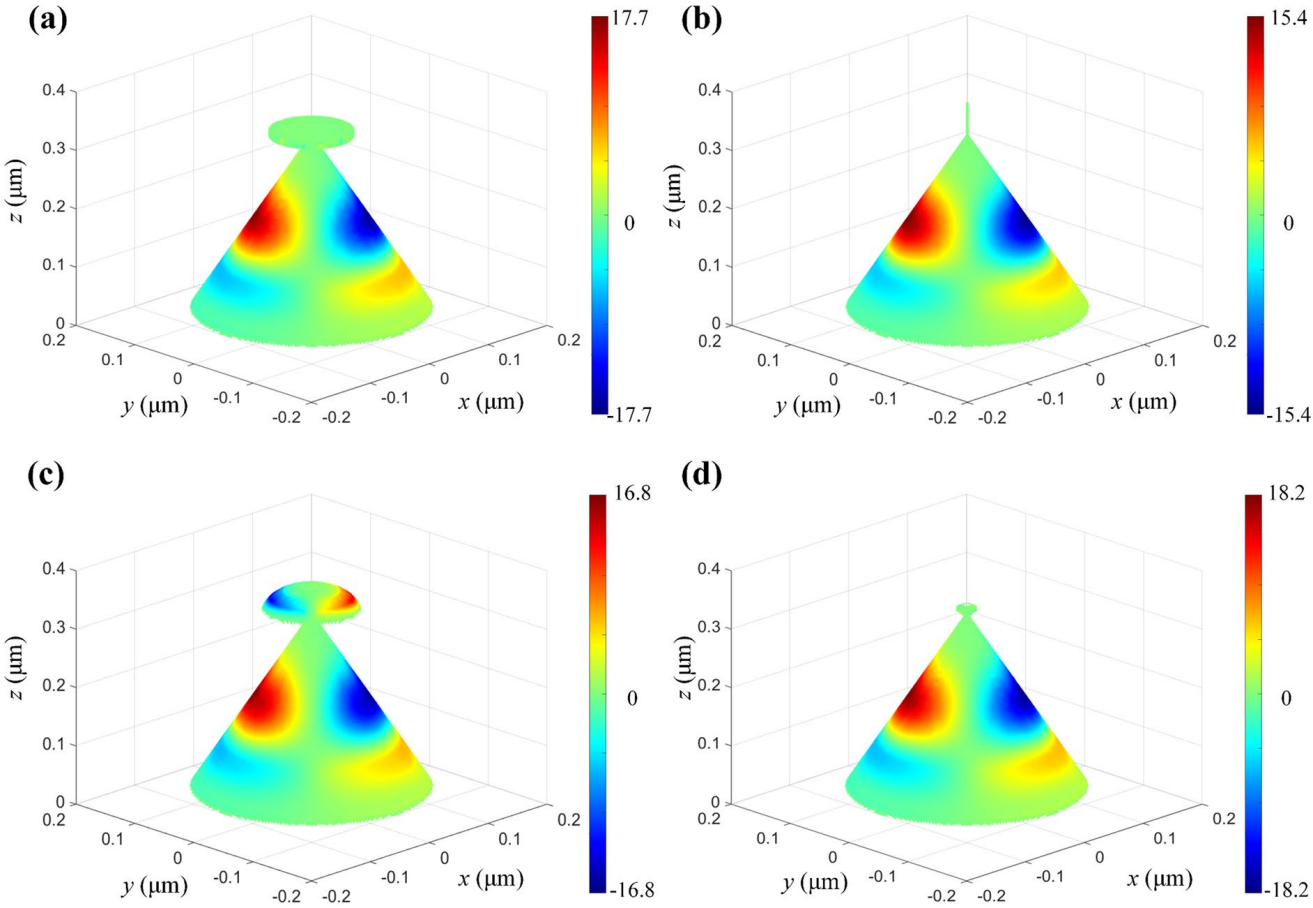
Typical 3D net-charge distribution of the complex NOAs with different nano-geometry and the apex attachment: (**a**) nano-disk; (**b**) nano-wire; (**c**) nano-semi-sphere; (**d**) nano-ball.

Continuously, several complex NOAs are constructed by directly configurating a featured nanostructure including a nano-disk with a radius of 50 nm and a height of 10 nm, a nano-wire with a radius of 1 nm and a height of 60 nm, a nano-semisphere with a radius of 50 nm, and a nano-ball with a radius of 10 nm. These components are positioned atop the apex of the conical NOA, which possesses a uniform bottom diameter of 280 nm and a top angle of 50°. The surface net-charge distribution of the NOAs is simulated for evaluating the influence of the apex nanostructure about the net-charge instantaneous density and fashion, as shown in Fig. 4. As demonstrated, the dipole net-charge distribution over the complex NOAs presents a similar standing wave pattern and density with a maximum value of ~ 16.5 unit. But, a relatively strong DNCS can be observed over the top nano-semisphere, as shown in Fig. 4c, which maybe a combined action of the Coulomb-like blockade and the quantum confinement^40–43^.

**Figure 5:**
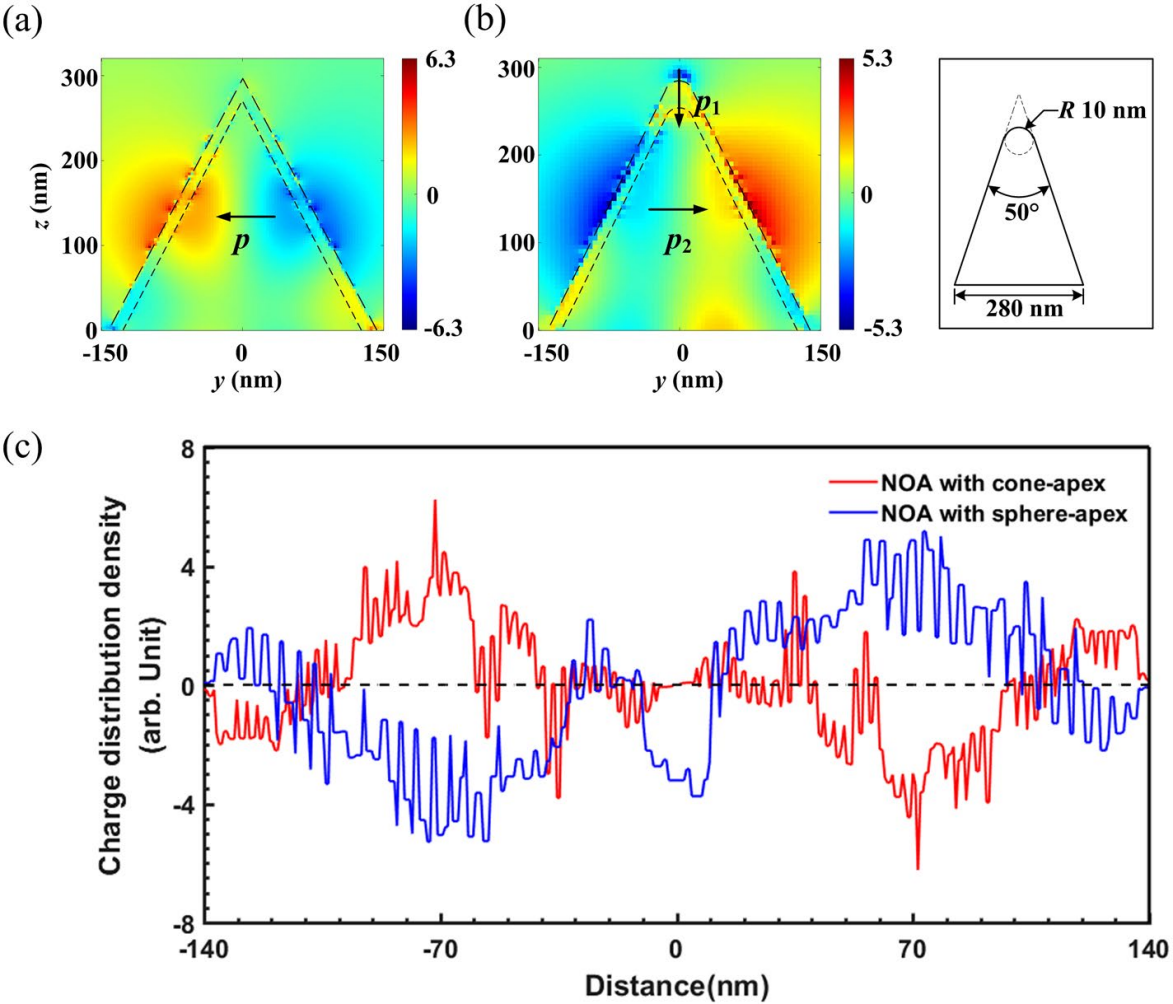
Comparison about the net-charge arrangement characters over the NOAs with different apex-appearance but the same lower frustum, whose top and bottom radius are 10 nm and 140 nm, respectively. The cross-sectional net-charge distribution density of the NOAs with (**a**) cone-apex and (**b**) sphere-apex in the *y–z* plane are also given, and a typical structure of the latter is displayed in the inset. The contour of the Au-NOA and the silicon NOA are also plotted along a long and a short dashed-line, respectively. (**c**) The net-charge distribution density over the surface of the NOAs with a cone-apex (blue line) and a sphere-apex (red line).

Moreover, a comparison about the net-charge distribution over the NOAs with a 30 nm gold film but different apex-appearance including the cone-apex and the sphere-apex, is also conducted. The cross-sectional net-charge arrangement characters of the NOAs are displayed in Fig. 5a,b. The contours of the Au-NOA and the silicon NOA are plotted in a long and a short dashed-line, respectively. A typical structural diagram of a NOA with a sphere-apex is also inserted. Currently, the NOA with a sphere-apex is shaped by a nano-sphere top with a radius of 10 nm and the same lower frustum as the NOA with a cone-apex having, i.e. the top and bottom radius of 10 nm and 140 nm and the top angle of 50°. Additionally, the net-charge distribution density over the surface of both the NOAs are also given, as demonstrated in Fig. 5c. The noise of the curves can be attributed to mismatching between the set simulated mesh and the contour of the NOAs. As shown, the main DNCS can also be observed in the area lower than the apex region over the opposite side, and still existing two horizontal dipole moments of ***p*** and ***p***_**2**_ in both the NOAs. Although the height of the central arc of the main DNCS is approximate, a larger net-charge distribution density with a maximum value of 6.23 unit around the apex can be found in the NOA with the cone-apex, whereas 5.28 unit corresponding to the sphere-apex. Besides, more “free electrons” can be accommodated over the apex region decorated by a nano-sphere top. For a typical QD, the increase in the specific surface area means a wider surface with corresponding more surface state for net-charges, following with the unoccupied energy level for more net-charges get into the QD. As the introduction of the NOA decorated by a nano-sphere top with a higher specific surface area, more surface state is provided for net-charge commendation, and thus more net-charge nano-converges within the apex region. Consequently, the net positive charges are then nano-converged toward the apex of the silicon NOA, and then a vertical dipole moment ***p***_**1**_ is additionally formed over the sphere-apex. More interesting, the orientation of ***p***_**1**_ will slightly deviate from the vertical direction in infrared region according to the simulations in a wavelength range from 400 nm to 3 μm, as shown in Fig. S4.

**Figure 6:**
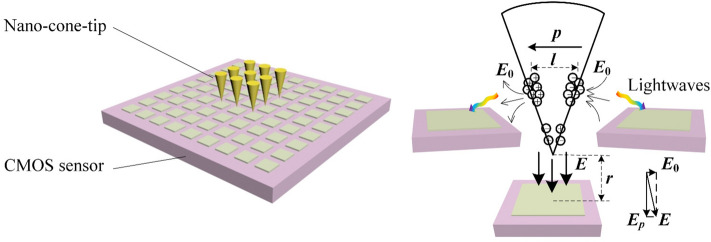
Detection scheme based on coupling an arrayed NOA and a CMOS sensor. The overall detection layout (left) and the schematic of a unit NOA as a dipole optical antenna irradiating lightwaves onto a single CMOS pixel.

It can be expected that a potential photosensitive application of the proposed NOAs will owe to a remarkable enhancement of incident lightwave collection efficiency for an arrayed complementary metal-oxide-semiconductor (CMOS) sensor, which means a highly efficient incident lightwave responding and resonantly converging or even focusing corresponding to only one or several CMOS pixels. The boosted detection architecture scheme based on the NOAs is displayed in Fig. 6. According to the simulations, a dipole net-charge nano-converging mainly takes place over the side surface surrounding the apex of a single NOA with a relatively large top angle, which will guide the stimulated surface waves towards CMOS sensors. A typical physical course is as follows. A dipole-typed optical antenna with a dipole moment $\user2{p = }fj(x,y,z)e{\varvec{l}}$, where *e* represents a single net-charge quantity, radiates outward transverse lightwaves with an electric-field *E*_*p*_. Meanwhile, another near-field lightwaves with an electric-field *E*_0_ will be superimposed with *E*_*p*_ and continuously illuminate the CMOS sensors coupled directly with the nano-tips mentioned. According to Fig. 5c, a higher net-charge distribution density over the cone-apex means a larger dipole moment, so as to generate a stronger electric-field *E*. Similarly, more net-charge converged over the sphere-apex will lead to a stronger CMOS photosensitive response.

### SNOM measurement

**Figure 7:**
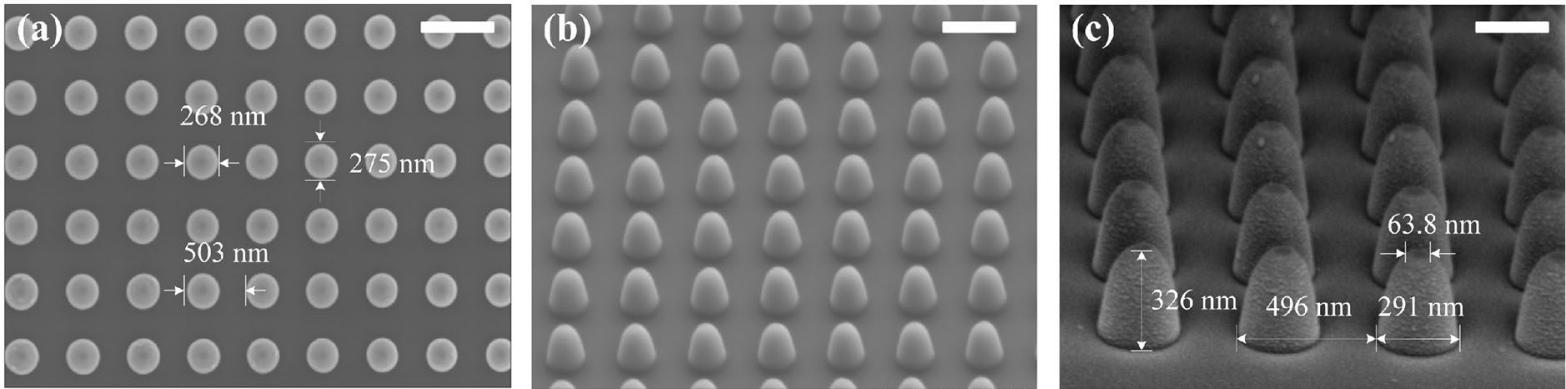
SEM images of an arrayed NOA before and after sputtering a gold film based on designed configuration such as each single NOA with a bottom diameter of 280 nm and a top angle of 50° and an arrangement period of 500 nm. (**a**) Top viewing of the fabricated silicon NOAs (scalar bar 550 nm); (**b**) 3D viewing of the same silicon NOAs (scalar bar 500 nm); (**c**) 3D viewing of the silicon Au NOAs (scalar bar 250 nm).

To actually explore the near-field lightwave characteristics, an arrayed NOA is fabricated over the surface of a n-type silicon substrate through electron-beam lithography (EBL, JBX 6300FS, JEOL) and inductivity coupled plasma etching (ICP, Oxford Plasma Pro System 100 ICP 380). The NOA is designed with a single bottom diameter of 280 nm, a top angle of 50°, and an arrangement period of 500 nm. And then a gold film with a thickness of 30 nm is further deposited over the silicon NOAs by magnetic sputtering (Kurt J. Lesker LAB 18). The scanning electron microscope (SEM, SU8220, Hitachi) photographs of the silicon NOA array before and after sputtering a gold film are shown in Fig. 7. A top viewing of the silicon NOAs with an average bottom diameter of ~ 270 nm is presented in Fig. 7a, where each selected single NOA demonstrates a slight bottom size variance by a typical horizontal-orientation 268 nm to another vertical-orientation 275 nm. Considering the etching accuracy of both the EBL and ICP and other intrinsic technological errors, the apex sharpness of the final NOAs with a radius of curvature of ~ 30.2 nm will generally be lower than those designed, as shown by NOAs with a typical nano-dome apex in Fig. 7b. After further depositing a gold film mentioned, the average NOA bottom diameter has expanded to a typical value of ~ 291 nm and the average height and the cross-sectional diameter of the apex facet ~ 326 nm and ~ 63.8 nm, respectively, as shown in Fig. 7c. So, the thickness of the gold film shaped over the surface of a single NOA is ~ 8 nm according to the measurement data, which is remarkably less than that deposited over the planar facet between adjacent NOAs.

**Figure 8:**
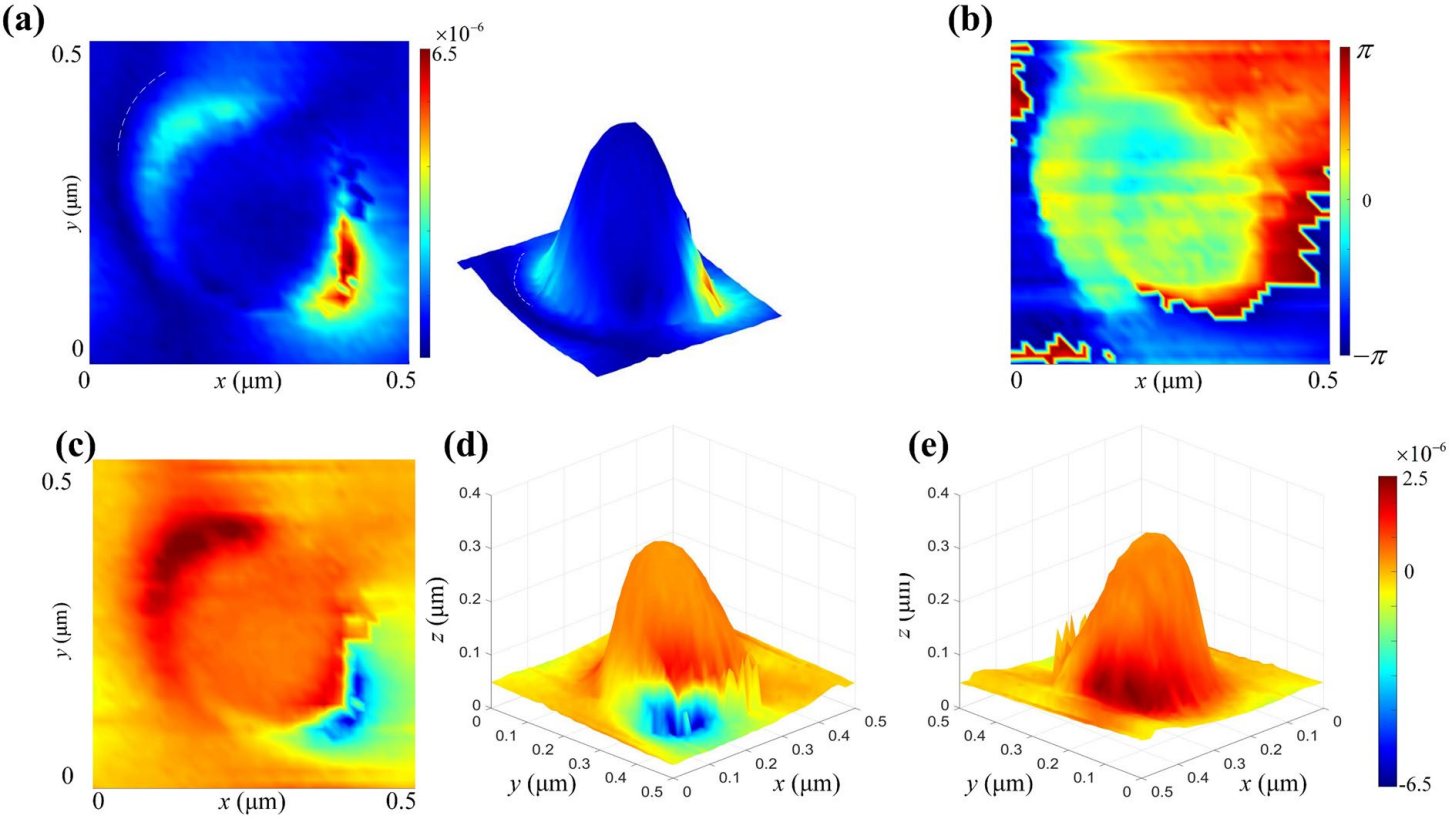
Microband-shaped near-field lightwave distribution over a single NOA obtained by SNOM measurement and the correlated surface net-charge instantaneous distribution reconstructed. (**a**) The top viewing and 3D presentation of the amplitude *S*_*7*_; (**b**) the top viewing of the phase *φ*_*7*_; (**c**) the top viewing of an instantaneous dipole net-charge distribution; (**d**) the 3D viewing of an instantaneous negative net-charge distribution illuminated by SNOM beams; (**e**) the 3D viewing of an instantaneous positive net-charge distribution.

The SNOM measurements using a scattering-type of SNOM (NeaSNOM, Neaspec GmbH Co.) in a reflectance mode with the same beam illumination condition, as indicated in Fig. S3 in the Supplementary Information, is performed to probe the near-field optical characteristics based on the near-field lightwave amplitude and phase data, and thus obtain the correlated surface net-charge instantaneous distribution map over the NOA according to Eq. (1), as illustrated in Fig. 8. Figure 8a,b show the visible near-field lightwave amplitude *S*_*7*_ and the phase *φ*_*7*_ of a single NOA, respectively, where the subscript 7 indicates Au film directly deposited over the silicon NOAs fabricated. As illustrated in Fig. 8a, there are two microband-shaped light spots colored by dark red with a largest value of 6.5 × 10^–6^ unit and light blue corresponding to a relatively small value of less than 3 × 10^–6^ unit according to the color-bar, respectively, which almost near the NOA bottom indicated by a dark annulus as labeled by a white dash line. As demonstrated in an inserted 3D viewing, they can be featured by an electric-field resonance enhancement localized at the lower right and the upper left nano-regions of the NOA covered entirely by SNOM beams during measurement. The near-field light intensity or amplitude corresponding to the left nano-region is relatively identical and then gradually weakened around. It should be noted that the near-field lightwaves excited by the SNOM beams are almost distributed over the surface of the Au NOA in a half-wavelength standing wave mode across the apex node of the NOA, which is very similar with the simulating prediction.

The reconstructed net-charge instantaneous distribution corresponding to the patterned near-field lightwaves is displayed in Fig. 8c,e, which intuitively describe a dipole-type instantaneously distribution of the net positive and negative charges over the side surface of a single NOA according to a top viewing and 3D presentations. As shown, the aggregated “free electrons” only instantaneously appear on the right side with a relatively large density according to a half-wavelength standing wave mode. Conversely, the counterpart with a relatively loose net positive charge arrangement are mainly situated on back side. The formed dipole net-charge distribution is basically consistent with the simulation shown in Fig. 2b. A top viewing of an instantaneous dipole net-charge distribution corresponding to actual microband-shaped light spots is re-constructed, which presents a similar morphology with the near-field lightwaves measured, as shown in Fig. 8c. Both 3D viewing of an instantaneous negative and positive net-charge distribution patterns are demonstrated in Fig. 8d,e, respectively. It should be noted that the asymmetrical dipole charge appearance is likely originated from several factors including: (1) the incident direction of the SNOM beams existing an obvious difference from the simulating setting of 45°, and (2) the relatively strong measurement beams already stimulating a tightly aggregating surface “free electrons”, and (3) the device existing ~ 30 nm inclining over the measurement bench.

**Figure 9:**
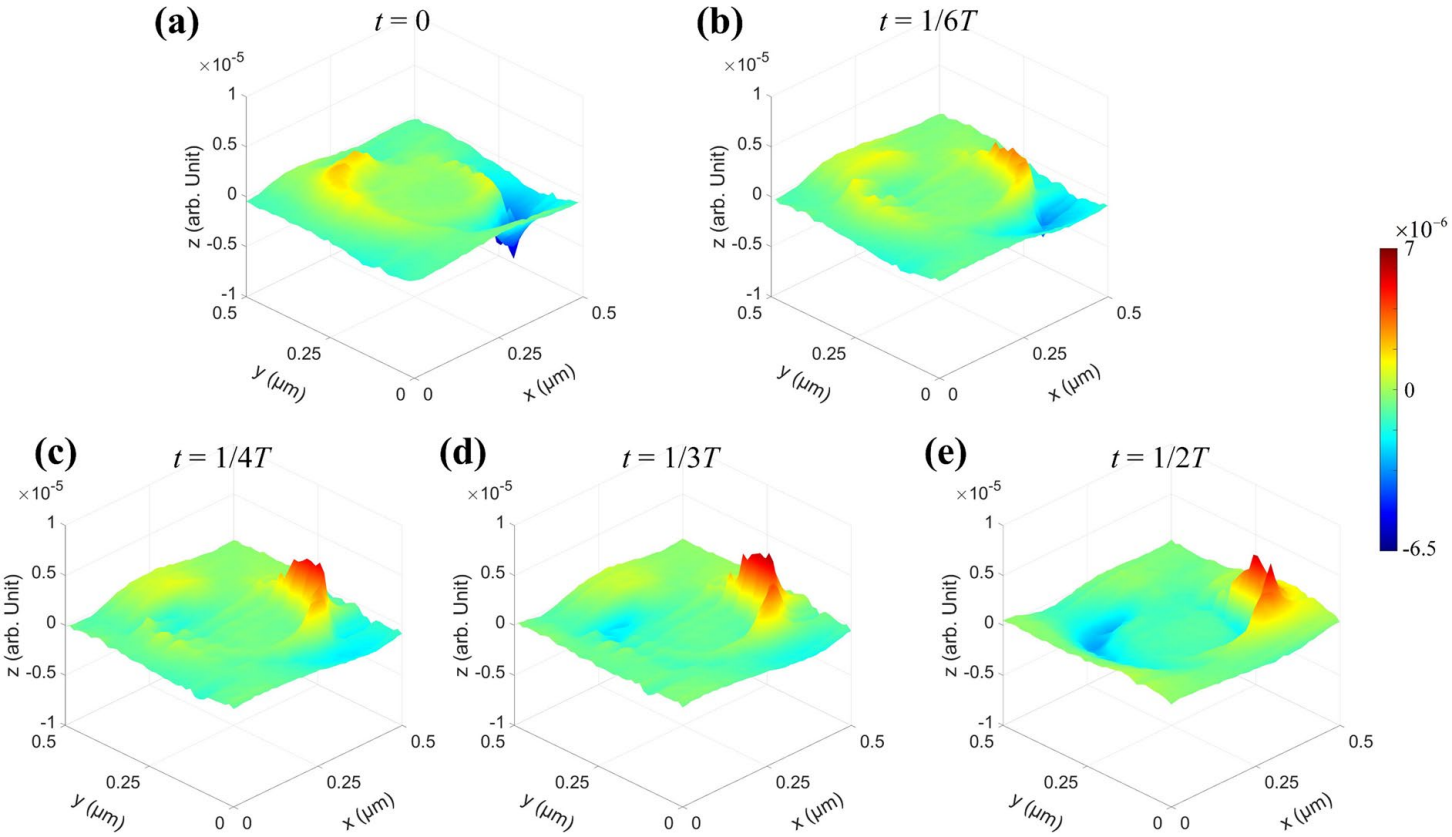
Time evolution of the net-charge instantaneous distribution *f *(*x, y, z, t*) over a single NOA based on the dipolar oscillation according to Eq. (2). A sequence of the typical net-charges distribution over a single NOA at several typical moments: (**a**) *t* = 0; (**b**) *t* = 1/6*T*; (**c**) *t* = 1/4*T*; (**d**) *t* = 1/3*T*; (**e**) *t* = 1/2*T*.

The excited near-field lightwave oscillation over the NOA can be represented based on Eq. (1) by the following equation of

2 $$ fj(x,y,z,t) = {\text{Re}} \left[Sj(x,y,z,\theta ,d)e^{{{\text{i}}\varphi_{j} (x,y,z,\theta ,d) - {\text{i2}{\pi }}t/T}} \right] $$

where *T* is the oscillation period. A series of snapshots of $f_{j} (x,y,z,t)$ within a half of *T* corresponding to a single NOA at selected moment such as *t* = 0, *t* = 1/6*T*, *t* = 1/4*T*, *t* = 1/3*T*, and *t* = 1/2*T*, are shown in Fig. 9, where *z*-axis represents the instantaneous surface net-charge distribution density. As illustrated, there existing a significant period variation of the dipole net-charge distribution density, and the maximum distribution density of “free electrons” already reaching ~ 5 × 10^–6^ units at initial moment shown in Fig. 9a, while the maximum density of the positive net-charges of ~ 7 × 10^–6^ units appears at *t* = 1/3*T* shown in Fig. 9d.

**Figure 10:**
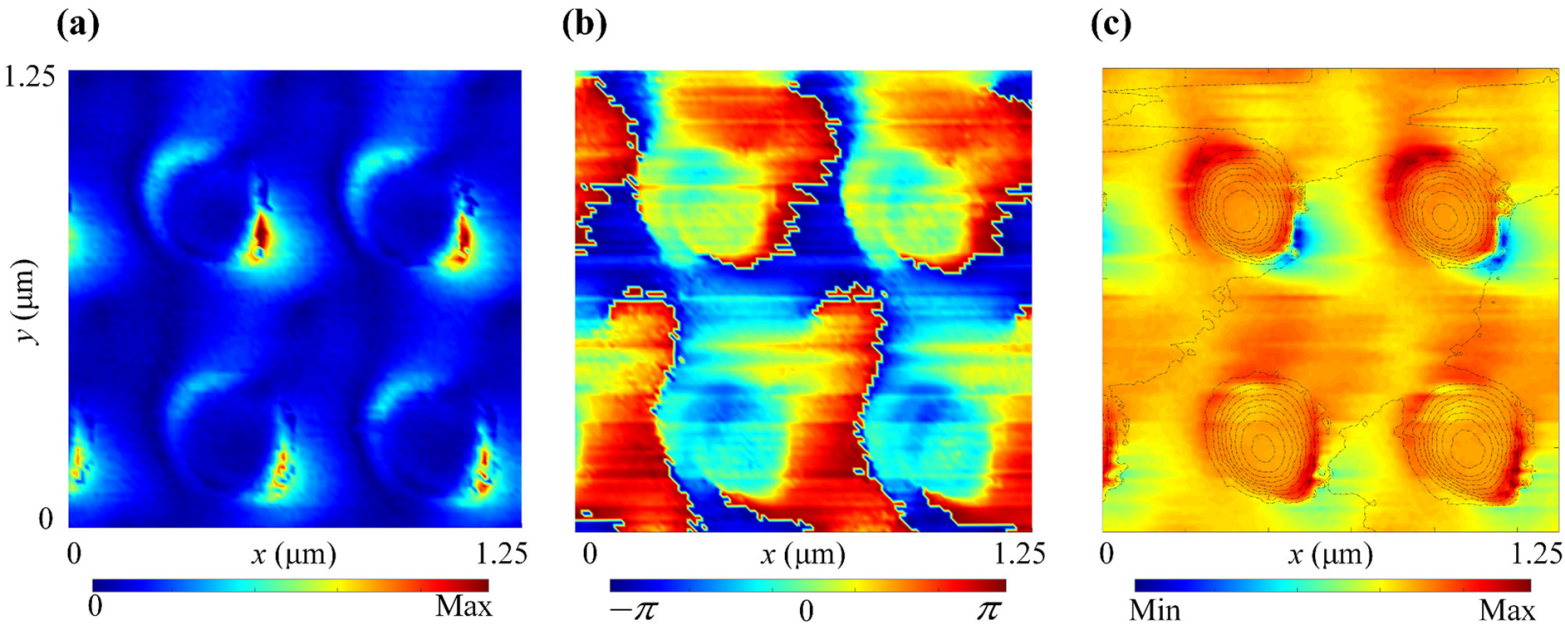
Near-field lightwave characteristics of an arrayed NOAs. (**a**) The amplitude *S*_*7*_; (**b**) the phase *φ*_*7*_; (**c**) a dipole of net-charge instantaneous distribution with the tip proximity effect.

An overview of the dipole net-charge instantaneous distribution over an arrayed Au NOA are given in Fig. 10. Both the amplitude and phase images are given by Fig. 10a,b, respectively. As shown, the shaped DNCSs are similar to that of a single Au NOA mentioned, but the upper NOAs present a greater amplitude due to the stronger illumination of SNOM beams. As shown in Fig. 10c, each NOA in the upper line nearly paralleled to the incident direction of SNOM beams, presents an obvious DNCSs nearing the NOA bottom and a relatively high density. However, a relatively weak positive net-charges still appears over the planar facet between NOAs. Moreover, the net-charges in a similar dipole mode over both adjacent NOAs with much weaker density. So, an obvious tip proximity effect can be observed, which means that the same species net-charges distributed over surrounding planar facet. Additionally, a weak dipole net-charge arrangement can be induced nearing the bottom boundary of each adjacent NOA. Furthermore, it can be predicted that this configuration will lead to a generation of localized resonant enhancement of the surface waves excited.

## Conclusions

The near-field lightwave behaviors of an arrayed silicon NOA covered by a common metal film, which are excited by measurement lasers with a central wavelength of 633 nm and then closely correlated with a dipole net-charge instantaneous distribution over the NOA surface, are carefully investigated. An obvious Coulomb-like blockade from the apex region of the NOA apparently influencing the net-charge converging over its side surface is clearly observed, which can also be predicted by the simulations according to the surface standing waves across the apex node corresponding to both the surface lightwaves and the surface net-charge density waves. The antinodes of the surface positive and negative net-charge instantaneous distribution are already pushed away the normal location owing to the Coulomb-like blockade effect of the apex, so as to present a distorted waveform different from the traditional standing wave mode. The tip proximity effect leading to a relatively weak nano-converging of the net-charges over surrounding planar facet and also adjacent NOAs, so as to generate a localized resonant enhancement of the surface waves, is also discovered. The research highlights the potential applications such as low-cost nano-lithography, super-high-intensity electron or light emission, and ultra-sensitive light detection through NOA.

## Methods

### Sample fabrication

The n-type silicon material is selected for forming an arrayed silicon NOA mainly by EBL and ICP etching.

At first, a photoresist array is fabricated over the silicon wafer via EBL (JBX 6300FS, JEOL). During the EBL exposure, the fine pattern is set to be nano-blocks (200 nm $\times$ 200 nm) with an arranged period of 500 nm. And then, a spin processing is conducted according to main parameters being set as 40 s and 4000 rpm for forming a layer of tackifier (AR 300-80) with a thickness of 20 nm for enhancing the adhesion between the wafer and the photoresist. After spin-coating, the sample is baked at 180 °C for 2 min. A negative photoresist (ma-N2403) with a thickness of 380 nm is fabricated according to main parameters being set as 40 s and 2000 rpm and afterwards baked at 90 °C for 1 min. Furthermore, an arrayed photoresist mask is defined by e-beam exposure, and then developed by tetramethylammonium hydroxide (TMAH) for 2 min to acquire a needed pattern over the surface of silicon substrate.

Secondly, the ICP etching (Oxford Plasma Pro System 100 ICP 380) is performed to form a nano-cone-tip array upon silicon wafer. The chemical reactions are conducted by SF6 for 5 sccm and C4F8 for 100 sccm. The main technological parameters include: the pressure of 12 mTorr, the ICP power of 1200 W, the RF power of 50 W, the reaction temperature of 10 ℃, and the etching time of 5.5 min.

Finally, a 30 nm thick gold film is deposited over the silicon nano-cone-tips using the magnetron sputtering (Kurt J. Lesker LAB 18), so as to construct an Au-NOA metasurface. Noting that a 20-nm-thick Ti layer is previously coated and thus directly contact to the silicon material for enhancing Au film’s adhesion over the tapered silicon NOA.

### SNOM measurements

Considering the significant sensitivity to the electric-field component outward from the surface of micro-nano-architectures, the scattering-type SNOM (NeaSNOM, Neaspec GmbH Co.) based on the atomic force microscope (AFM) system is adopted to measure the net-charge distribution density over the facet of the NOAs. The experimental set-up is briefly described below. The *p*-polarized incident beams with a central wavelength of ~ 633 nm is incident obliquely along the diagonal with an incident angle of 45° upon a horizontal facet, which is the same as that shown in Fig. 1a. During measurements, the incident beams is focused via a parabolic mirror onto both the sample and the platinum AFM probe oscillated vertically, which is also applied as a scattering source and its tip-scattered light is modified according to the near-field lightwave properties of the sample below the tip. And then, the tip-scattered light is demodulated at the *n-*th harmonics of the tapping frequency yielding background-free images. To fully filter out the background signal, *n* = 3 is currently chosen in this work. Finally, both the amplitude and phase of the tip-scattered light, delivering the information about the near-field electric-component signals, are recovered through an all-optical interferometric detection^44^.

### Supplementary Information

Supplementary Information.

## Data Availability

The datasets generated and/or analysed during the current study are not publicly available but are available from the corresponding author on reasonable request.
