# Supplementary material for: Research on the localized lightfield features of metallic nano-cone-tip optical antenna via investigating near-field lightwave and correlated net-charge distribution
Source: Sci Rep. 2023 Dec 12;13:22002. doi: 10.1038/s41598-023-49097-y (PMC10716382; doi:10.1038/s41598-023-49097-y)
Supplement: Supplementary file 1 — Supplementary Information. [file 41598_2023_49097_MOESM1_ESM.docx]

Supplementary Material for

“Research on the Localized Lightfield Features of Metallic Nano-Cone-Tip Optical Antenna via Investigating Near-Field Lightwave and Correlated

Net-Charge Distribution”

Taige Liu^1,2^, Jiashuo Shi^1,2^, Shenghua Duan ^1,2^, Wuyang Ji^1,2^, Zhe Wang^1,2^ and Xinyu Zhang^1,2,*^

^1^National Key Laboratory of Science & Technology on Multispectral Information Processing, Huazhong University of Science & Technology, Wuhan 430074, China

^2^School of automation & artificial intelligence, Huazhong university of science & technology,

Wuhan 430074, China

*[x_yzhang@hust.edu.cn](mailto:x_yzhang@hust.edu.cn)

The more supplementary details of the dipole net-charge distribution over the nano-cone-tip with the same bottom diameter of 280 nm but different top angle, including the amplitude and phase images, are displayed in figure S1. Besides, the patterned net-charge rearrangement of the tip with different structural parameters of *θ=*50° and *θ=*30° are further exhibited. As shown in the amplitude images, it presents a gradually strengthened amplitude, and the maximum value of the amplitude gets more nearly to the apex node with the decreased top angle.

As for the top viewing of the dipole net-charge rearrangement, the distance between the apex node and the central arc of the dipole central arc of a main DNCS is of an increasing sequence of ~180 nm and ~196 nm and ~216 nm and ~279 nm and ~423 nm according to the dimensioned data of the upper long dashed and lower short dashed circles. The increasing distance is consistent with the conclusion of the greater Coulomb-like blockade effect as the apex being sharper obtained in the main text.

Here, the enlarged viewings of the patterned net-charge over the apex region are also provided in figure S1, whose intensity drops during the process of shrinking top angle. In particular, the maximum distribution density of the net positive charge and aggregated “free electrons” over the apex region is slightly lower than that of the nano-cone-tips with adjacent parameters when *θ=*50°, but presents a closer instantaneous net-charge distribution to the apex, which is basically consistent with the phenomenon in the main text.

In order to avoid repetition, the similar simulation details of the nano-cone-tip with different structural material and apex nano-geometry are not listed here.

**Figure S1.** Supplementary details of the dipole net-charge distribution over the nano-cone-tip with different structural parameters: (a) *d*=280 nm, *θ=*60°, (b) *d*=280 nm, *θ=*50°, (c) *d*=280 nm, *θ=*40°, (d) *d*=280 nm, *θ=*30°, (e) *d*=280 nm, *θ=*20°. The amplitude and phase images of each featured nano-cone-tip are listed in detail, and then the top and enlarged viewing of the surface net-charge instantaneous distribution can be given according to Eq. 1 in the main text.

The DNCS describes the area constraining a large amount of net-charge, and thus with the significantly high net-charge distribution density, which corresponds to the peak of the stimulated surface wave. However, the apex region with obvious Coulomb-like blockade is the area beyond the DNCS and with extremely low density, which is considered as lower than 0.1 unit. Figure S2 describes the surface net-charge distribution density variation along the diagonal for all six conditions in the Fig. 1 and 2 in the manuscript is plotted, and the density curves over the apex region is provided in Fig. S3. As illustrated in Fig. S2, the distance between the peaks, distribute opposite the 0 point representing the position of the DNCS, are ~125.4 nm, ~115.4 nm, ~132.6 nm, ~122.0 nm, ~112.0 nm and ~125.4 nm, respectively. The distance between the peaks is in consistence with the region within the labelled dashed lines in Fig.1 and Fig. 2 of the manuscript. Besides, the density along the upper edge of the DNCS reaches ~1.5 unit according to Fig. 1 and Fig. 2 of the manuscript. As for the defined quasi quantum dot, the area with supremely low distribution density can be clearly observed in Fig. S3. The bottom diameters of the apex with strong Coulomb-like blockade are ~3.3 nm, ~15.0 nm, ~16.7 nm, ~6.7 nm, ~6.7 nm and ~10.0 nm, respectively. The typical structural parameters are basically lower than the de Broglie wavelength, which is ~10nm in semiconductor. The diameters demonstrate that the Coulomb-like blockade is enhanced with the decreasing top angle and results in the larger bottom diameters of the apex region over the NOA, which is consistent with the conclusion in the manuscript.


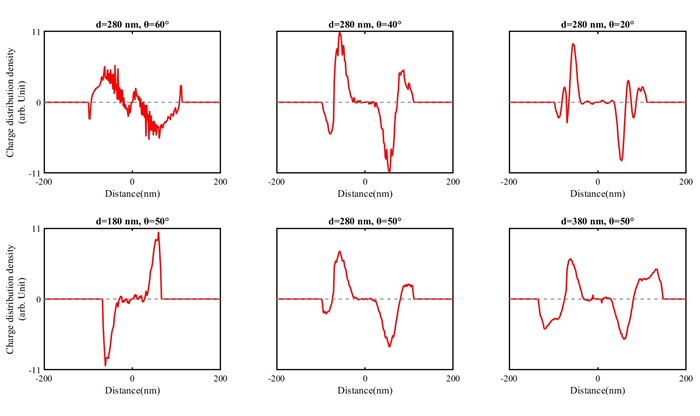


**Figure S2.** Curves of the surface net-charge distribution density along the diagonal for all six kinds of structural parameters in Fig. 1 and Fig. 2 of the manucript. The distribution density of 0 unit is also plotted in gray dotted line.


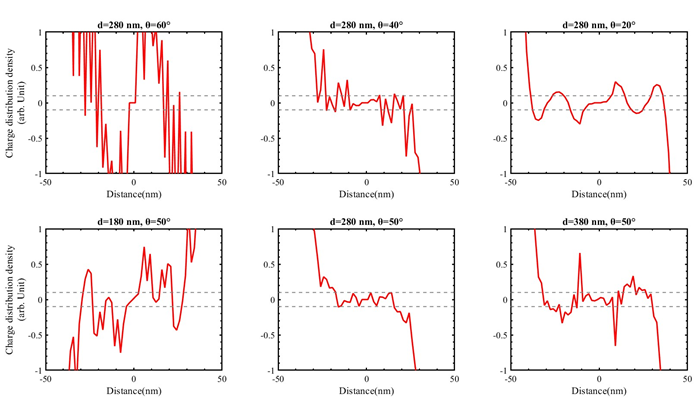


**Figure S3.** Surface net-charge distribution density curve over the apex region. The reference lines with constant values of -0.1 and 0.1 unit are plotted in the gray dashed lines.

The net-charge instantaneous arrangement over the NOA decorated with sphere-apex and coated with a 30 nm gold film under different incident wavelength is quantitatively simulated, and the typical results at specific wavelength are displayed in Fig. S4. The positive charge appears at the apex region of the golden NOA with sphere-apex, while the negative charge is induced in the area between the gold film and the silicon NOA, and the distribution density of the two charge is almost numerically equal at 400 nm. At the lower part of the proposed NOA, there is an alternating distribution of positive and negative charge, but the distribution density is extremely low and not symmetric along the geometric central axis of the NOA. At 453 nm, the situation of the net-charge distribution is in opposite, where the negative charge appears at the apex region and the positive charge are induced around. However, the distribution density of negative charge is close to three times than which of positive charge. Besides, the distribution density of net-charge at the lower part is approximately the same as the case of 400 nm.


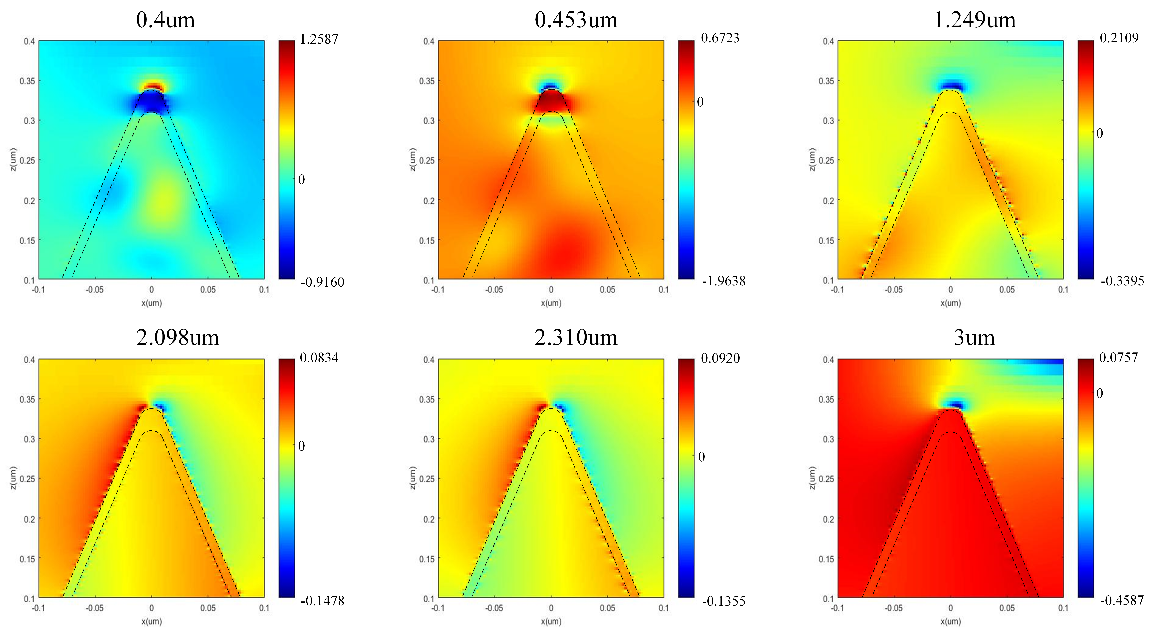


**Figure S4.** Typical simulation results about the dipole net-charge distribution over the nano-cone-tip decorated with sphere-apex irradiated by lightwave with different incident wavelength from 400 nm to 3 μm.

As for the infrared region, the negative charge at the tip of the gold film tends to shift to the right, and the quantity of positive charge gradually increase within the apex region. Moreover, the charge at the area below the apex gradually distributes axis-symmetrically. As the wavelength increases at 2.098 μm, the positive charge with a higher distribution density begin to appear at the NOA apex, but are still far less than the distribution density of negative charge. At 2.310 μm, the distribution density of positive charge at the apex reach the highest. Then, when the incident wavelength reaches 3 μm, the negative charge distribution density at the apex exceeds one order of magnitude compared to the positive charge. Besides, there is almost no negative charge in the lower area of NOA.


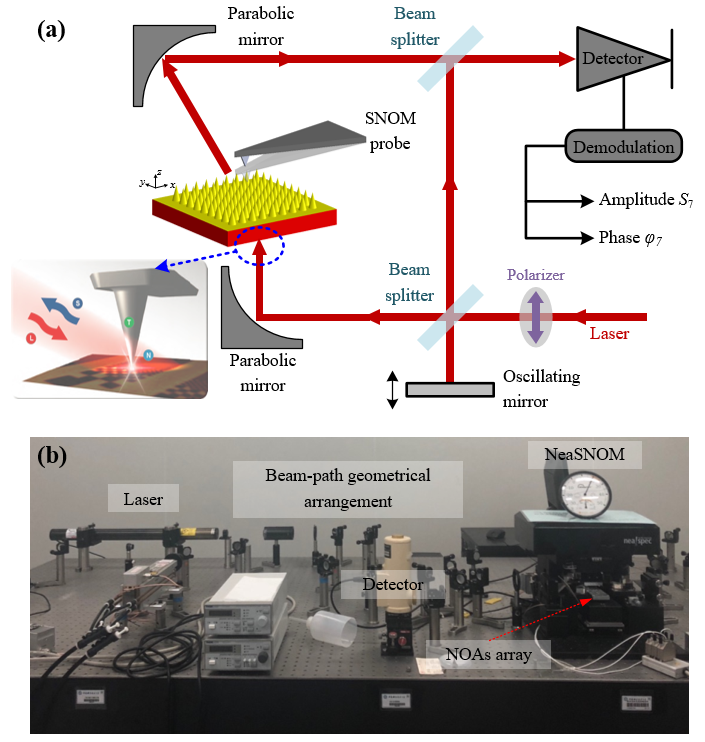


**Figure S5.** Experimental set-ups of SNOM measurement. (a) Measuring optical path schematic and (b) testing configuration for performing SNOM measurement based on an arrayed NOA covered by a gold film with a thickness of 30 nm. Note that Fig. S5(a) only describe the experimental beam path but not the incident direction. Actually, the visible laser is incident downwards and obliquely along the diagonal with an incident angle of 45° upon a horizontal facet of the proposed arrayed NOA.

The basic experimental setting for performing SNOM measurement based on an arrayed NOA covered by a gold film with a thickness of 30 nm, is illustrated in Fig. S5. After passing through the experimental beam-path geometrical arrangement, a visible laser beam with a central wavelength of ~633 nm is incident obliquely along the diagonal with an incident angle of 45° upon a horizontal facet of the proposed arrayed NOA. During the measurements, the incident beams is focused via a parabolic mirror onto both the sample and the platinum AFM probe oscillated vertically. Besides, an evanescent field is generated over the gold-coated sample surface via incident laser. Then, the focal spot is utilized to scan the evanescent field generated over the sample surface. The evanescent field can be affected by the surrounding NOAs over the sample surface, resulting in the scattered light (labeled with blue arrow in the subplot of Fig. S5(a)), carrying the near-field optical information, received in the far-field of the detection area. Then, the tip-scattered light is demodulated at the *n*-th harmonics of the tapping frequency yielding background-free images. To fully filter out the background signal, n = 3 is currently chosen in this work. Finally, both the amplitude and phase of the tip-scattered light, delivering the information about the near-field electric-component signals, are demodulated through an all-optical interferometric detection.
